# Solitary Metastasis in the Mediastinal Lymph Node After Radical Nephrectomy for Clear Cell Renal Cell Carcinoma: A Case Report and Literature Review

**DOI:** 10.3389/fonc.2020.593142

**Published:** 2020-12-17

**Authors:** Hang Lin, Heng Zhang, Yuanda Cheng, Chunfang Zhang

**Affiliations:** ^1^ Department of Thoracic Surgery, Xiangya Hospital, Central South University, Changsha, China; ^2^ Human Engineering Research Center for Pulmonary Nodules Precise Diagnosis and Treatment, Xiangya Hospital, Central South University, Changsha, China; ^3^ National Clinical Research Center for Geriatric Disorders, Xiangya Hospital, Central South University, Changsha, China; ^4^ Xiangya Lung Cancer Center, Xiangya Hospital, Central South University, Changsha, China

**Keywords:** renal cell carcinoma, clear cell renal cell carcinoma, solitary metastasis, posterior mediastinal lymph node, surgical resection

## Abstract

**Background:**

Renal cell carcinoma can metastasize to virtually any anatomical site throughout the body, especially the lung, bone, lymph nodes, liver, and brain. However, it is extremely rare for renal cell carcinoma to metastasize solely to the mediastinal lymph node more than 15 years after radical nephrectomy.

**Case Presentation:**

The case we present here is that of a 50-year-old Chinese male with an isolated posterior mediastinal lymph node metastasis of clear cell renal cell carcinoma 16 years after radical nephrectomy. However, based on imaging examination, the mass was clinically misdiagnosed as Castleman’s disease before operation. Following surgical excision of the mass, it was finally judged to be a metastasis from clear cell renal cell carcinoma according to the patient’s medical history and immunohistochemical findings. Currently, there is no clinical or radiological finding the recurrence of metastasis after 10 months of follow-up.

**Conclusion:**

We report a case of solitary metastasis in the posterior mediastinal lymph node 16 years after radical nephrectomy for clear cell renal cell carcinoma. Given the long disease-free interval between primary renal cell carcinoma to isolated mediastinal lymph node metastasis, it is important to conduct a lifelong regular follow-up, including thoracic computed tomography. In addition, surgical resection remains the best method of treatment for mediastinal lymph node metastases from clear cell renal cell carcinoma if the metastatic lesion is limited.

## Background

Kidney cancer is one of the most common types of cancer in men and women, accounting for approximately 4.2% of all new cancer cases in the United States in 2019 (1). As the leading form of kidney cancer, renal cell carcinoma (RCC) represents approximately 90% of all malignancies of the kidney (2). Due to the aggressive nature of RCC, 20%–30% of patients have synchronous metastases at the time of initial diagnosis, and 20-40% of patients develop metachronous metastases after nephrectomy (3). The lung, bone, lymph node, liver, and brain are the most frequent sites of metastases from RCC (4). In most cases, mediastinal lymph node (MLN) metastases of RCC are usually accompanied by lung metastases (5). In contrast, in the absence of lung metastases, isolated MLN metastasis is rare (5). Herein, we report a case of solitary metastasis to a posterior MLN 16 years after radical nephrectomy for clear cell renal cell carcinoma (CCRCC) and summarize previous reports of similar diseases. The case report is of great value for the understanding of the unusual metastatic pattern of RCC.

## Case Presentation

In November 2019, a 50-year-old Chinese male was admitted to the hospital with intermittent cough and expectoration, which had lasted for more than one month. He was diagnosed as having a right renal tumor measuring 3.8 cm in diameter 16 years earlier, and he had undergone right radical nephrectomy. Postoperative pathological examination revealed Fuhrman grade II CCRCC (p T1a N0 M0). After surgery, he did not have a regular follow-up.

**Figure 1:**
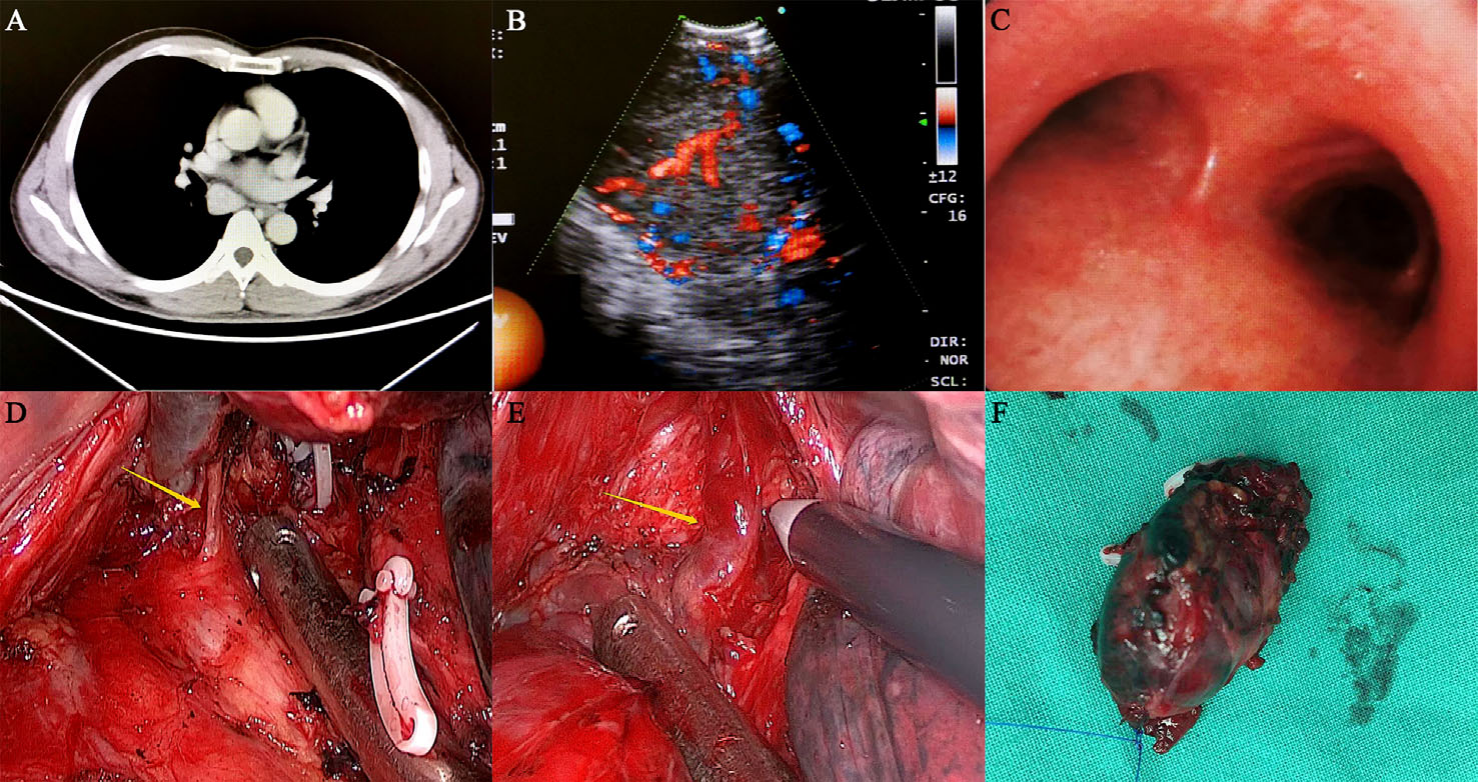
Preoperative examination and intraoperative status of the patient. **(A)** The thoracic contrast-enhanced CT showed that the mass was located in the right posterior mediastinum, and its size was approximately 40*35 mm. The CT value of the plain scan was approximately 46 HU, and the CT value was approximately 130 HU after enhancement. **(B**, **C)** Endobronchial ultrasonography showed that a posterior mediastinal mass was located far from the carina with a complete capsule, clear margin and adequate blood supply. **(D**, **E)** During the operation, the lesion was located under the carina and surrounded by several abnormally large bronchial arteries, which had an extremely abundant blood supply. **(F)** The mass was completely removed and was approximately 3.5 cm in size.

Physical examination and laboratory examination showed no significant findings. Thoracic contrast-enhanced computed tomography (CT) showed that a 40*35 mm mass was located in the posterior mediastinum, and it was clinically diagnosed as Castleman’s disease (**Figure 1A**). Endobronchial ultrasonography showed that the posterior mediastinal mass was located far from the carina with a complete capsule, clear margin, and adequate blood supply (**Figures 1B, C**). However, other enlarged lymph nodes, local recurrence or distant metastases were not noted. Owing to the deep location and abundant vascularity of the mass, as well as the risk of bleeding, endobronchial ultrasound-guided transbronchial needle aspiration (EBUS-TBNA) was not performed.

**Figure 2:**
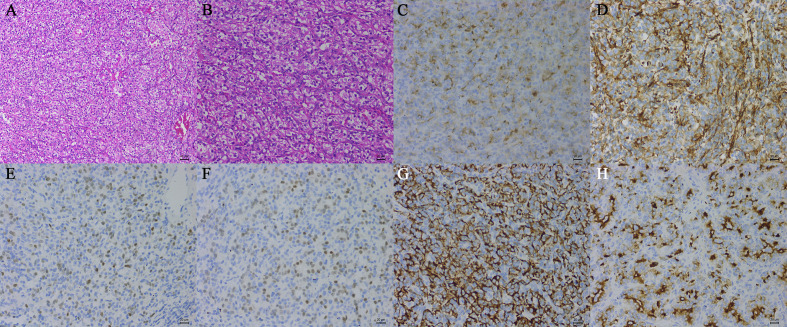
Histopathological examination of tissue samples (hematoxylin-eosin, original magnification, A: ×100, B: ×200). The tumor cells were positive for CD10 **(C)**, vimentin **(D)**, PAX-2 **(E)**, PAX-8 **(F)**, CK-Pan **(G)**, and the RCC marker **(H)** (immunohistochemistry, original magnification, ×200).

On November 21, 2019, the patient underwent video-assisted thoracoscopic surgery under general anesthesia. During the operation, the lesion was located under the carina with five abnormally large bronchial arteries (**Figures 1D, E**). No other swollen lymph nodes were observed. Fortunately, by gradually mobilizing the surrounding tissue of the mass and carefully ligating these bronchial arteries with the Hem-o-lok clips, the mass was completely removed (**Figure 1F**). Postoperative pathological examination indicated that the mass was a right mediastinal malignant tumor (**Figures 2A, B**). Immunohistochemical staining showed that the tumor cells were positive for cluster of differentiation 10 (CD10), vimentin, paired box 2 (PAX-2), paired box 8 (PAX-8), CK-Pan, and RCC markers but negative for cytokeratin 7 (CK 7), CgA, CD56, and synaptophysin (Syn) (**Figures 2C–H**). According to the patient’s medical history and immunohistochemical findings, it was finally judged to be a metastasis from CCRCC. The patient had an uneventful postoperative recovery, and he underwent no further therapy after the operation. Currently, there is no clinical or radiological finding the recurrence of metastasis after 10 months of follow-up.

## Discussion

RCC, a urological malignant tumor, frequently metastasizes at any time after nephrectomy (6). Currently, RCC can metastasize to virtually any anatomical site throughout the body, especially the lung, bone, lymph nodes, liver, and brain (4). In most cases, MLN metastases of RCC are usually accompanied by lung metastases (5). In contrast, in the absence of lung metastases, isolated MLN metastasis is clinically uncommon (5). In 1965, Arkless reported MLN metastases in 11 of the 152 RCC patients, all of whom presented concomitant lung metastases (7). In 1981, Saitoh reported that only 75 of the 1451 RCC patients suffered MLN metastases, and over 90% of these patients had concomitant lung metastases (8). The disease-free interval from diagnosis as primary RCC to reoccurrence or distant metastasis varies from several months to many years. Thompson et al. reported that the longest disease-free interval was that of a RCC patient found in the pancreas metastasis 32.7 years after radical nephrectomy (9). In this case, the RCC patient experienced posterior MLN metastasis 16 years after radical nephrectomy. This is exceedingly rare in the study of MLN metastases and underscores the importance of a lifelong regular follow-up in RCC patients, including thoracic CT.

A PubMed search of reports published in the literature using the medical terms “renal cell carcinoma” and “mediastinal lymph node metastasis” yielded 80 articles. We analyzed the data of 36 RCC patients who had an isolated MLN metastasis after radical nephrectomy from these reports (**Table 1**). Among the 36 RCC patients, 30 were men (83.3%), five were women (13.9%) and one was unknown (2.8%). The median age at metastasis was 59 years (range, 38–81 years), and the median interval from nephrectomy to isolated MLN metastasis was 2.75 years (range, 0–23.3 years). Twenty-six RCC patients underwent surgical resection of isolated MLN metastasis, with a median survival time from metastasectomy to the last follow-up of 2.1 years (range, 0.2–9 years). One patient received immunotherapy, and the remaining patients were not mentioned in the literature.

**Table 1:** Cases published in the literature on metastatic RCC to the MLN solely.

Author	Year	Total cases	Sex	Age at metastasis (y)	Pathological type	Time from nephrectomy to isolated MLN metastasis (y)	Treatment approach	Time from metastasectomy to last follow-up time (y)	Outcome at last follow-up
Slaton et al. (10)	1997	1	N/A	N/A	RCC	0	Surgery	N/A	N/A
Takanami et al. (11)	1998	1	M	50	RCC	1.0	Surgery	6.0	Alive
Niikura et al. (12)	1999	1	M	62	RCC	19.0	Surgery	N/A	N/A
Fritscher-Ravens et al. (13)	2000	1	M	72	CCRCC	7.0	Immunochemotherapy	N/A	N/A
Whitson et al. (14)	2008	9	7 M, 2 F	40–81	RCC	0.5–23.3	Surgery	0.2–3.2	8 Alive, 1 Died
Kanzaki et al. (5)	2009	2	1 M, 1 F	58–60	RCC	2.0–13.0	Surgery	1.8–7.0	2 Alive
Val-bernal et al. (15)	2018	9	9 M	44–74	CCRCC	0–4.6	N/A	N/A	N/A
Sponholz et al. (16)	2020	12	10M, 2 F	38–70	8 CCRCC, 1 Papillary RCC 3 unknown	0–14.3	Surgery	0.17–9.0	7 Alive, 4 Died, 1 N/A

M, male; F, female; RCC, renal cell carcinoma; CCRCC, clear cell renal cell carcinoma; N/A, not available.

### Metastatic Pathways

**Figure 3:**
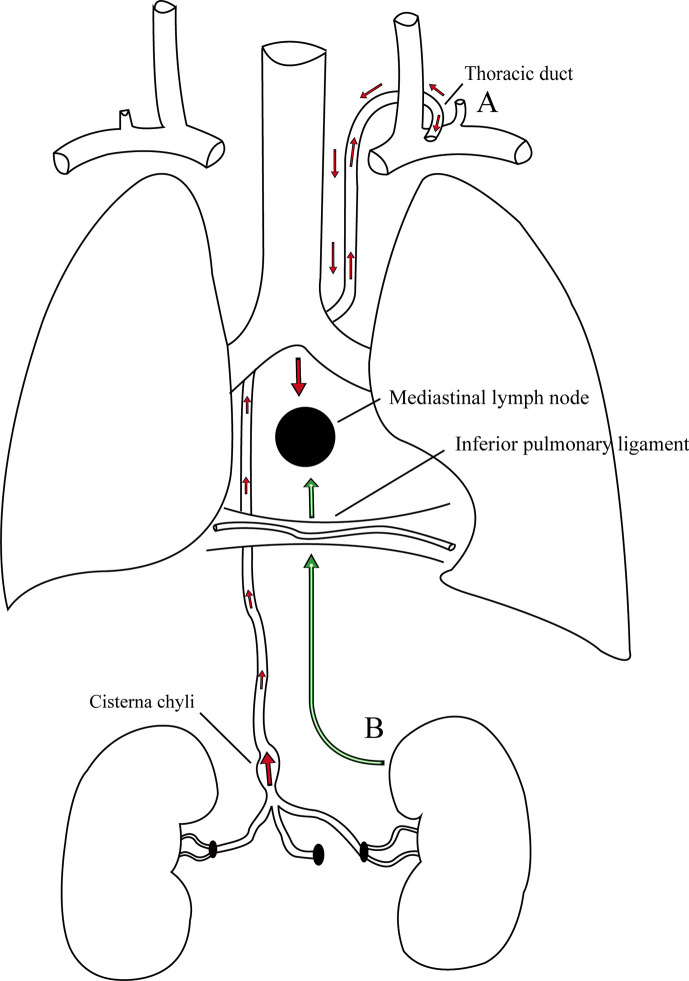
There are two possible pathways of metastasis in this case. **(A)** The cancer cells initially enter into the thoracic duct along the abdominal lymphatic vessels, then retrograde from the thoracic duct to the bronchomediastinal trunks due to incompetent valves, and finally reach the MLNs. **(B)** The cancer cells usually travel along the retroperitoneal lymphatic vessels into the lymphatic vessels in the inferior pulmonary ligaments and eventually reach the MLNs.

Interestingly, in this case, we found that the enlargement of the posterior MLN was a solitary metastatic lesion without any involvement of other lymph nodes and organs. However, the mechanism of MLN metastasis from primary RCC is not completely known. To date, there are two possible metastatic pathways that may explain the phenomenon (**Figure 3**). McLoud et al. reported that the pathway of metastasis is closely related to the thoracic duct. The cancer cells initially enter into the thoracic duct along the abdominal lymphatic vessels (17). However, if the valves in the lymphatics are incompetent, these cancer cells will retrograde from the thoracic duct to the bronchomediastinal trunks and finally reach the MLNs (17). Rosenberger et al. found that 10%–15% of the examined patients experienced regurgitation into the MLNs during lymphangiography because of incompetent valves (18). In addition, Wright described another pathway of metastasis in which cancer cells usually travel along the retroperitoneal lymphatic vessels into the inferior pulmonary ligament and eventually reach the MLNs (19).

### Important Examinations

#### Thoracic CT

In general, thoracic contrast-enhanced CT is a useful imaging technique for determining the location and appearance of the lesion and the extent of surgical resection. The radiological diagnosis of MLN involvement depends on the morphological characteristics appearing in the CT scan, mainly the increase in lymph node size (20). Normally, lymph nodes do not exceed 10 mm in diameter (20). If a MLN larger than 2 cm is detected, it is considered to be caused by metastatic malignancy (21). However, the use of size to identify MLN metastasis in RCC is limited (21). Metastatic lymph nodes less than 10 mm have been reported in approximately 5% of RCC patients (21). Due to the abundant vascularity of the lymph nodes, the lesion demonstrates distinct enhancement during enhanced CT scans (20). In addition, primary lymphadenopathy is difficult to distinguish from enlarged lymph nodes caused by metastatic diseases (20). In this case, the CT scans showed a round, well-circumscribed mass without calcifications in the posterior mediastinum, and the contrast-enhanced CT scan displayed distinct enhancement of the mass (**Figure 1A**). The CT features of our patient were consistent with those reported in the literature.

#### EBUS-TBNA

EBUS-TBNA plays an important role in evaluating MLN involvement in patients with RCC (15). As a minimally invasive modality, EBUS-TBNA usually carries out fine-needle aspiration biopsy of MLNs under the guidance of ultrasound through the bronchoscope. Mediastinal lymphadenopathy under EBUS mainly manifests as a lymph node with an increase in size as well as irregularity, inhomogeneity, hypervascularity, and hyperechoic echotexture (15). At present, the sensitivity and overall accuracy of EBUS-TBNA are 87% and 88%, respectively (22). The incidence of complications with EBUS-TBNA was relatively low, ranging from 1.23% to 1.44% (15). EBUS-TBNA is recognized as a safe and feasible method for the diagnosis of MLN metastases from RCC and may be considered a preferred auxiliary examination for these patients (22, 23). Regretfully, in this case, EBUS-TBNA was not performed in the RCC patient due to the deep location and abundant vascularity of the mass, as well as the risk of bleeding.

#### Histology

Histology is the mainstay for the diagnosis of metastatic RCC. It is well known that RCCs are a heterogeneous group of malignancies with many different histological subtypes (24). CCRCC is recognized as the most predominant histological subtype, accounting for approximately 70-75% of all RCC cases (25). It has been reported that more than 90% of patients with metastatic RCC are previously diagnosed with CCRCC (20). The cytological characteristics of metastatic CCRCC are similar to those of primary CCRCC. Macroscopically, the section of CCRCC appears typically golden yellow because the cells are rich in lipids. Areas of necrosis and hemorrhage are frequent in higher-grade tumors (25). Microscopically, CCRCCs have diverse growth patterns. Most low-grade tumors show acinar patterns, while high-grade tumors may tend to display solid, pseudopapillary, rhabdoid, or sarcomatoid patterns (26). These cells have abundant clear cytoplasm with a low nuclear-to-cytoplasmic ratio and often contain microvesicles (25, 27). The features of the large round nuclei vary with the tumor grade in CCRCC. For high-grade tumors, the nuclei are markedly irregular with prominent nucleoli, whereas the nuclei of low-grade tumors are slightly irregular with inconspicuous nucleoli (27). Papillary RCC (types 1 and 2) and chromophobe RCC occur in approximately 15%–20% of RCC patients (25). Crucially, there are significant differences in the biological behavior and prognosis among different subtypes of RCC, which makes correct histological diagnosis extremely essential.

#### Immunohistochemistry

The distinction between metastatic CCRCC and primary mediastinal lymphadenopathy with similar morphological features may be difficult in histological diagnosis. Immunohistochemistry is of great value in diagnosing metastatic CCRCC. The following markers are known to help in the diagnosis of metastatic CCRCC. Vimentin is a mesenchymal marker that is diffusely expressed in most subtypes of primary RCC, especially CCRCC and papillary RCC (28). Although vimentin is also noted in several other malignant tumors, it can help to narrow down the differential diagnosis of metastatic RCC (28). PAX-2, which is one of the nine members of the paired box gene family, is a renal cell lineage transcription factor (28, 29). It is highly expressed in most RCC subtypes, such as CCRCC, papillary RCC, and collecting duct RCC (28). Although PAX-2 has been recognized as a putative marker of metastatic CCRCC in many studies, the expression of PAX-2 in high-grade tumors is significantly lower than that in low-grade tumors, especially in CCRCC (28). Several studies reported the PAX-2 immunoreactivity in metastatic CCRCCs, and the results showed a sensitivity of 77%–85% and a specificity of 90%–97% (29, 30). PAX-8 is a transcription factor from the same family as PAX-2 (28, 31). It is strongly positive in most RCC subtypes, such as CCRCC, papillary RCC, and collecting duct RCC (28). McKenney et al. studied the PAX-8 immunoreactivity in metastatic CCRCCs, and the results showed the sensitivity of 94% and the specificity of 88% (32). PAX-2 and PAX-8 are confirmed as useful markers for metastatic RCC, regardless of histological subtypes (29, 31, 32). The RCC marker, which is a monoclonal antibody, is directed against an antigen found in the brush border of normal proximal renal tubules in the kidney (28, 33). Almost all CCRCC and papillary RCC are strongly positive for the RCC marker (28). McGregor et al. reported the immunoreactivity of the RCC marker in primary and metastatic RCCs, and the data demonstrated that the sensitivity of the RCC marker was 80% (122/153) in primary RCCs (84% in CCRCC) and 67% (42/63) in metastatic RCCs (33). Some studies showed that the sensitivity and specificity of the RCC marker in metastatic CCRCC ranged from 44% (7/16) to 70% (19/27) and 52% to 100%, respectively (30, 34). Obviously, owing to the low sensitivity, the RCC marker may be useful for differential diagnosis in some cases. CD10 is a cell-membrane-associated neutral endopeptidase that can hydrolyze peptide bonds (34, 35). It has a strong expression in CCRCC, papillary RCC, and Xp11 translocation RCC (28). Some studies revealed that the sensitivity of CD10 in metastatic CCRCC ranged from 83% (5/6) to 100% (16/16) (34, 35). However, the expression of CD10 is also widely noted in a large variety of other neoplasms, including pancreatic adenocarcinomas, colonic adenocarcinomas, ovarian carcinomas, and so on (28). Due to the low specificity, the use of CD10 as a marker for metastatic CCRCC requires careful consideration. Carbonic anhydrase (CA) IX, a tumor hypoxia marker, is involved in tumor aggressiveness and progression that is widely expressed in patients with CCRCC (36). Some studies recently showed that it might be used in the diagnosis of kidney tumors because it differentiates between CCRCC and the other subtypes of RCC (37, 38). Regrettably, there are no markers specific to CCRCC. In this case, the tumor cells were positive for vimentin, PAX-2, PAX-8, CD10, Ki67, CK-Pan, and RCC markers but negative for CK 7, CgA, CD56, and Syn (**Figures 2C–H**).

### Differential Diagnosis

The low incidence of isolated MLN metastasis and the lack of specificity of clinical symptoms and imaging manifestations in CCRCC often make it difficult to differentiate from primary mediastinal lymphadenopathy, which can lead to preoperative misdiagnosis. Even so, it is essential to distinguish metastatic MLNs from other mediastinal lymphadenopathies in patients with mediastinal lesions and a previous history of RCC (13, 39). The differential diagnosis of MLN enlargement includes lymphoma, sarcoidosis, tuberculosis, histoplasmosis, and solid malignancy (40). In this case, the patient was misdiagnosed with Castleman’s disease before the operation. Castleman’s disease is a rare benign lymphoproliferative disease, also known as hemangiomatous lymphoid hamartoma (41). It mainly occurs in the thorax, especially in the mediastinum, followed by the neck and abdomen (42). The chest CT findings of this disease are similar to those of metastatic CCRCC, and both of them present hypervascular tumors. We seriously reflected on the main reason for this misdiagnosis, and we thought that the mediastinal mass was not associated with the patient’s previous medical history of CCRCC before the operation and simply considered them to be two solitary tumors. We thought that the diagnosis of the mediastinal mass could not completely rely on the imaging examination, and we should have fully combined the patient’s medical history, clinical symptoms and physical examination and then made a correct diagnosis after careful identification.

### Effective Strategy

In terms of overall survival, surgical resection is a valid treatment for RCC patients with limited metastasis (43). Conversely, RCC is significantly resistant to conventional systemic chemotherapy and radiotherapy, and only a few patients have a complete response to immunotherapy (44). Some studies have reported that in many metastatic sites including the pancreas, liver, and lung, and the survival benefits after the surgical resection of an isolated metastasis were significant. Staehler et al. evaluated the efficacy of liver metastasectomy in metastatic RCC (45). They reported that 68 of 88 RCC patients considered to have liver metastases underwent liver metastasectomy, with a median survival of 142 months, and the remaining patients had no liver metastasectomy, with a median survival of 27 months (45). Zerbi et al. analyzed the prognosis of pancreatic metastases in 36 RCC patients. They revealed that the 5-year survival rates were 88% for 23 surgically removed patients and 47% for 13 nonsurgically removed patients (46). Kanzaki et al. summarized the long-term results of surgical resection for lung metastasis from RCC. The results showed that the 3-, 5-, and 10-year survival rates of 48 RCC patients were 60%, 47%, and 18%, respectively (47). Whitson et al. assessed the outcomes after metastasectomy of RCC patients with isolated MLN metastases. The outcomes showed that the 1-, 3-, and 5-year survival rates were 100%, 80%, and 80%, respectively, and RCC patients who underwent isolated MLN metastasectomy had a survival advantage as compared with other patients having stage IV disease (14). Recently, Sponholz et al. demonstrated the long-term outcomes after resection of isolated thoracic lymph node metastases of RCC. The study presented that 14 RCC patients were included in the long-term follow-up with a median follow-up time of 35.5 months (range, 2-108 months), and the 1-, 3-, and 5-year survival rates for 15 RCC patients were 93%, 73%, and 73%, respectively (16). Surprisingly, this is the largest cohort of RCC with isolated thoracic lymph node metastases in the published literature (16). In this case, the patient underwent complete resection of the MLN metastasis with video-assisted thoracoscopy, and he has undergone no further therapy since the operation. Currently, there is no clinical or radiological finding the recurrence of metastasis after 10 months of follow-up.

## Conclusion

Isolated MLN metastasis of RCC is clinically uncommon. We report a case of solitary metastasis in the posterior MLN 16 years after radical nephrectomy for CCRCC. Given the long disease-free interval between primary RCC to isolated MLN metastasis, it is important to conduct a regular oncologic follow-up, especially thoracic CT. Furthermore, immunohistochemistry is an effective tool in distinguishing metastatic CCRCC from primary mediastinal lymphadenopathy with similar morphological features. Surgical resection remains the best method of treatment for MLN metastases from CCRCC if the metastatic lesion is limited.

## Data Availability Statement

The original contributions presented in the study are included in the article/supplementary materials. Further inquiries can be directed to the corresponding authors.

## Ethics Statement

Written informed consent was obtained from the patient for publication of any potentially identifiable images or data included in this article.

## Author Contributions 

HZ and YC performed the surgery in this case. HL retrieved clinical information, drafted the manuscript, and performed the literature review. CZ and YC conceived, designed, and supervised this article. All authors contributed to the article and approved the submitted version.

## Conflict of Interest

The authors declare that the research was conducted in the absence of any commercial or financial relationships that could be construed as a potential conflict of interest.
